# Effects of low lead exposure on sperm quality and sperm DNA methylation in adult men

**DOI:** 10.1186/s13578-021-00665-7

**Published:** 2021-07-30

**Authors:** Tiancheng Zhang, Yan Fei Ru, Bin Wu, Haiyan Dong, Liang Chen, Jufen Zheng, Jianhui Li, Xin Wang, Zhikai Wang, Xuemei Wang, Xiaorong Shen, Jun Wu, Jun Qian, Maohua Miao, Yihua Gu, Huijuan Shi

**Affiliations:** 1 NHC Key Lab of Reproduction Regulation, Shanghai Institute for Biomedical and Pharmaceutical Technologies, Fudan University, Shanghai, China; 2 Key Laboratory of Growth Regulation and Translational Research of Zhejiang Province, School of Life Sciences, Westlake University, Hangzhou, 310024 Zhejiang China; 3 Shanghai Kelin Institute of clinical bioinformatics, Shanghai, China

**Keywords:** Low lead, Sperm motility, DNA methylation, Calcium and lead interaction, Calcium homeostasis regulation pathway

## Abstract

**Instruction:**

Lead (Pb) exposure is a risk factor for male infertility, but the epigenetic changes in sperm DNAattributable to lead exposure is poorly defined.

**Methods:**

In this study, we investigated whether low Pb exposure (<  10 µg/dL) affects the sperm quality. Blood, urine, and semen samples of 297 men of childbearing age were analyzed for all relevant parameters. Based on the blood Pb level (BLL), participants were allocated to RL (0–2.5 µg/dL), RM (2.5–5 µg/dL), and RH (5–10 µg/dL) groups. The 5-methylcytosine and 5-hydroxymethylcytosine patterns in the sperm DNA were identified using methylated DNA immunoprecipitation and hydroxymethylated DNA immunoprecipitation sequencing.

**Results:**

The non-progressive motility (NP) was significantly increased and associated with global hypomethylation of sperm DNA in the RH group compared with the RL group, indicating that aberrant sperm methylation due to low Pb exposure is possibly associated with reduced sperm motility. The hypomethylated promoter regions were primarily enriched in the calcium (Ca) homeostasis pathway. Further, the interaction between Ca and Pb was associated with sperm rapid progressive motility and asthenospermia risk, although no significant methylation abnormality was observed in those with BLL  <  5 µg/dL. When BLL was  >  5 µg/dL or when predicting NP, no significant Pb–Ca interaction was observed.

**Discussion:**

Overall, our results indicate that aberrant DNA methylation of the Ca homeostasis pathway, induced by low Pb exposure, is the potential cause for reduced sperm velocity.

**Supplementary Information:**

The online version contains supplementary material available at 10.1186/s13578-021-00665-7.

## Introduction

Research suggests that the effect of Pb exposure on the sperm quality, an important indicator of male fertility, is dose-dependent [1]. According to the Centers for Disease Control (CDC), a blood Pb level (BLL) of  >  10 µg/dL is a public health concern [2]. However, studies have shown that the Pb exposure in men with an average BLL of  <  10 µg/dL causes reduced sperm quality. For an example, a study by Jurasovic et al. [3] in 123 Croatian volunteers with a median BLL of 5.7 µg/dL (range: 2.5–14.9 µg/dL) reported that the BLL is negatively correlated with sperm motility and morphology. Similar results were observed in another study involving 240 volunteers with a median BLL of 4.92 µg/dL (range: 1.13–14.91 µg/dL) [4]. However, it is well known that a wide concentration range may result in heteroscedasticity of data, where the variance increases with increasing concentrations [5]. The analytical results of investigations examining the biological effects of low Pb exposure may be inaccurate, especially at a lower concentration range, if individuals exposed with a high BLL are included. Notably, previous studies have failed to exclude individuals with a high BLL [3, 4]. In this study, we explored the potential effects of low Pb exposure on the sperm quality. Only men with a BLL  <  10 µg/dL were included.

Large-scale DNA methylation reprogramming occurs during gamete formation and early embryonic period in humans, and the process is susceptible to environmental factors [6, 7]. However, previous relevant research has mainly focused on determining the effect of environmental Pb exposure on DNA methylation in peripheral blood [8]. Christine et al. [9] demonstrated that maternal Pb exposure (mean BLL: 5 µg/dL) can cause changes in peripheral blood DNA methylation of important genes involved in fetal development, in line with the results of a study by Shaowei et al. [10]. In addition, DNA methylation is one of the key mechanisms to maintain Ca homeostasis. A study had shown that the Ca homeostasis pathway regulated by DNA methylation is related to the risk of coronary heart disease [11]. The maintenance of Ca homeostasis is the key factor to ensure sperm motility [12, 13]. However, only a few studies have investigated the possible effects of low-level parental Pb exposure on human sperm DNA methylation and Ca homeostasis pathway regulated by DNA methylation.

In this population-based study, we determined whether the level of Pb exposure represented by a BLL of  <  10 µg/dL is an independent risk factor for poor sperm quality. In addition, we used sperm DNA methylation data, quantified through methylated DNA immunoprecipitation (MeDIP) and hydroxymethylated DNA immunoprecipitation (hMeDIP) sequencing, to explore the mechanism associating low Pb exposure with sperm quality.

## Materials and methods

### Study participants

This cross-sectional study involving men of childbearing age living in the Sandu County, Guizhou Province, China, was conducted from September 2011 to April 2012 by using random cluster sampling.

After excluding those with left testis and right testis volume of  <  12 mL, sperm density  <  1  ×  10^6^/mL abnormal semen agglutination, and blood Pb concentration  >  10 µg/dL, a total of 297 men were included in this study, with an average age of 32.71 years. The severity of smoking habit was recorded as the number of cigarettes smoked per day, and 271 participants reported of daily alcohol intake. The study was approved by the Ethics Committee and Institutional Review Board of Shanghai Institute of Planned Parenthood Research. All the study participants provided written informed consent.

### Semen analysis

All participants had to abstain from sex for 2–7 days before providing the semen samples. The samples were collected by masturbation into 25-mL sterile polystyrene jars and analyzed within 1 h of ejaculation. Computer-assisted sperm analysis (CASA) (WLJY-9000, Beijing, China) was performed to obtain sperm concentration, viability, and motility after the liquefaction of sample at the clinics, in accordance with the World Health Organization (WHO) guidelines (fifth edition) [14].

An aliquot of semen was centrifuged at 3000 rpm for 5 min. The supernatant was frozen without preservatives and temporarily stored at − 20 °C. All the samples were shipped to the laboratory at the Shanghai Institute of Planned Parenthood Research (Shanghai, China) on dry ice and stored at − 80 °C for the Ca, Mg, and Zn concentration assays.

Total Zn, Ca, and Mg concentrations in semen were measured through flame atomic absorption spectrometry by using a BH5500S device (Bohui Innovation Technology Co., Ltd., Beijing, China). The calibration solution in the determinations was provided by the Bohui Company, and accuracy of the calibration solution was set according to the national standard material for Zn (GBW08620), Ca [GBW(E)080118], and Mg [GBW(E)080126].

### Blood analysis

The nursing staff obtained 5 mL of venous blood into anticoagulant tubes from participants’ upper limbs. Similar to Zn, Ca, and Mg assays, the Pb and cadmium (Cd) levels in the blood were determined using a graphite furnace atomic absorption spectrophotometer (same as the detection scheme of trace elements in seminal plasma). The labeling curve was drawn by using the standard solution of Pb and Cd provided by China National Research Center for Labeling Substances. Serum FSH, LH, and total testosterone were determined using an electrochemiluminescence immunoassay with kits provided by Roche Diagnostics GmbH (Mannheim, Germany).

### Urine analysis

A single-spot urine sample was collected from all the participants during their visit to the clinic. The urine samples were analyzed at the collaborative laboratory of East China University of Science and Technology (Shanghai, China). Urinary concentrations of total BPA, DEHP, MEHP and DBP were measured using modified high-performance liquid chromatography. The same method was used to detect Zn, Ca, and Mg in both urine and semen samples.

#### MeDIP and hMeDIP sequencing

Sperm DNA of nine men (three men randomly selected from each of the RL, RM, and RH groups) aged 20–40 years were submitted for MeDIP and hMeDIP sequencing. These men did not have a history of smoking or alcohol intake, their blood Cd concentration was  <  1 µg/dL, and BPA, DEHP, MEHP, and DBP concentrations were  <  1 µg/µmolCr. The sperm DNA was extracted from samples using the DNeasy Blood and Tissue kit (Qiagen) according to the manufacturer’s instructions. Agarose gel electrophoresis was used to detect the integrity of genome, Nano drop (Thermo Scientific) was used to detect DNA purity (OD 260/280 ratio), and the DNA concentration was measured using Qubit 2.0 fluorometer (Life Technologies). The extracted DNA was fragmented using the Covaris sonication system, and sequencing libraries were prepared from the 5-μg fragments of genomic DNA. End repair, A base addition, and adaptor ligation steps were performed using the Illumina’s Single-End DNA sample preparation kit. Adaptor-ligated DNA was immunoprecipitated by anti-5mC and anti-5hmC by using a commercial antibody. The immunoprecipitated DNA was purified and then applied for 50-bp single-end sequencing on the Illumina Hiseq2500 platform.

### Bioinformatic analysis

All sequencing data passed initial quality checks for base composition through FASTQC v0.10.0 (15). For each individual,  ~  20 million reads were generated and mapped onto hg19 by using BWA [16]. After removing duplicates, we used macs2 [17] to call peaks with the following parameters: -t $path-f BAM-g hs-B-n $path_suffix-broad-broad-cutoff 0.1-fix-bimodal-extsize 300. Then, R package ChIP-seq was used to evaluate the quality of samples, and the diffbind package was used to evaluate DMRs between the samples [18, 19]. The screening threshold of DMRs was the log_2_(fold change)  >  1, FDR  <  0.05, and the differential binding region was annotated by CHIPseeker [20]. The R package clusterprofiler [20] was used to annotate DMRs in the promoter region of the gene and to perform Gene Ontology (GO) [21], Kyoto Encyclopedia of Genes and Genomes (KEGG) [22], and Ingenuity Pathway Analysis (IPA) analysis. The gene where the differential promoter region was located was used to create a heatmap by using the pheatmap Package.

## Statistical analysis

Demographic characteristics and blood, semen, and urine parameters were statistically analyzed. The sample frequency was counted by classified variables, and the mean value, standard deviation were counted by numerical variables. Logistic regression analysis was used to classify variables and linear regression analysis was used to classify numerical variables, to explore factors affecting male fertility. By including the interaction term Pb and Ca in sperm in the full model, we investigate whether relationships of Pb and response changed based on the value of Ca in sperm. P values  <  0.05 were considered statistically significant. Apart from considering significant interaction effect, we compare two nested models that whether included interaction term or not by ANOVAs test to select the best fit. The R package stats was used to implement the regression analysis and ggpubr was used to display the results. All statistical analysis were performed using the R 3.6.3 package.

## Results

### Description of study participants

Blood samples were obtained from all the participants for assessing hormone, Pb, and Cd levels. The mean hormone levels were as follows: follicle stimulating hormone (FSH): 5.14 IU/L; luteinizing hormone (LH): 5.26 U/L; and testosterone: 5.55 ng/mL. The mean BLL was 3.15 µg/dL, and the mean blood Cd level was 5.87 µg/dL. Urine analysis revealed the following plastifier levels (mean values): bisphenol A (BPA): 1.71 µg/µmolCr; dioctyl phthalate (DEHP): 6.09 µg/µmolCr; dibutyl phthalate (DBP): 2.39 µg/µmolCr; and mono-2-ethylhexyl phthalate (MEHP): 3.22 µg/µmolCr. The mean urine zinc (Zn), Ca, and magnesium (Mg) levels were 10.34, 3.06, and 1.98 mmol/L, respectively. Semen samples were evaluated for rapid progressive motility (mean, 27.35%), slow progressive motility (14.01%), non-progressive motility (13.38%), sperm concentration (54.54  ×  10^6^/mL), and sperm viability (64.26%). Seminal plasma samples were analyzed for Zn (mean, 2.42 mmol/L), Ca (9.52 mmol/L), and Mg (4.48 mmol/L). Testicular scale was used to measure the testicular volume; the mean left testicular volume was 22.79 mL and right testicular volume was 22.6 mL. The participants were allocated to three groups based on the BLL: RH group (BLL: 5–10 µg/dL; n  =  68), RM group (BLL: 2.5–5 µg/dL; n  =  91), and RL group (BLL: 0–2.5 µg/dL; n =  138; Table 1).

**Table 1:** Characteristics of study participants

	Groups	All participant [mean (sd)]	RL group [mean (sd)]	RM group [mean (sd)]	RH group [mean (sd)]
Statics	N	297	138	91	68
Age	297	32.71 (6.92)	32.52 (6.98)	32.91 (7.09)	32.82 (6.64)
Alcohol (yes)	271	91.2%	92.8%	91.1%	88.2%
Smoke (cigarettes per day)	297	9.77 (8.85)	9.66 (9.68)	9.3 (8.09)	10.62 (8.08)
Blood Pb (µg/dL)	297	3.15 (2.39)	1.09 (0.79)	3.68 (0.69)	6.60 (1.47)
Blood Cd (µg/dL)	297	5.87 (6.44)	3.9 (4.85)	7.05 (6.76)	8.3 (7.61)
Left testicular volume (mL)	297	22.79 (5.69)	22.2 (5.51)	23.97 (6.16)	22.43 (5.23)
Right testicular volume (mL)	297	22.6 (5.81)	22.17 (5.51)	23.24 (6.64)	22.59 (5.19)
Rapid progressive motility %	297	27.35 (14.97)	27.4 (15.07)	27.91 (15.69)	26.48 (13.94)
Slow progressive motility %	297	14.01 (6.62)	13.43 (6.51)	14.27 (6.58)	14.85 (6.86)
Non-progressive motility %	297	13.38 (8.87)	12.26 (8.14)	13.11 (8.16)	16.02 (10.63)
Sperm concentration (10^6^/mL)	296	54.54 (43.37)	49.61 (39.87)	60.96 (49.5)	55.9 (40.69)
Sperm viability %	183	64.26 (18.91)	64.07 (18.39)	63.95 (20.33)	65.01 (18.75)
FSH (IU/L)	294	5.14 (2.47)	5.17 (2.7)	5.02 (2.51)	5.26 (1.9)
LH (U/L)	294	5.26 (2.52)	5.42 (2.86)	5.13 (2.3)	5.14 (2.02)
T (ng/mL)	294	5.55 (1.92)	5.39 (1.84)	5.49 (1.73)	5.96 (2.26)
BPA (µg/µmolcr)	297	1.71 (9.52)	1.13 (2.84)	0.89 (1.94)	3.99 (19.29)
DEHP (µg/µmolcr)	297	6.09 (30.38)	6.74 (35.79)	7.67 (31.53)	2.66 (10.27)
DBP (µg/µmolcr)	297	2.39 (7.52)	3.02 (9.41)	2.26 (6.55)	1.27 (2.96)
MEHP (µg/µmolcr)	297	3.22 (9.02)	4.02 (11.67)	3.03 (6.16)	1.87 (5.15)
Zn in Semen (mmol/L)	297	2.42 (1.27)	2.31 (1.3)	2.54 (1.28)	2.48 (1.19)
Ca in Semen (mmol/L)	297	9.52 (3.83)	9.27 (3.89)	9.59 (3.73)	9.96 (3.85)
Mg in Semen (mmol/L)	297	4.48 (3.01)	4.18 (2.92)	4.66 (2.91)	4.82 (3.3)
Uzn in Urine (mmol/L)	297	10.34 (7.81)	10.45 (8.31)	10.25 (6.94)	10.25 (7.96)
Uca in Urine (mmol/L)	297	3.06 (2.23)	3.16 (2.36)	2.93 (2.07)	3.02 (2.22)
Umg in Urine (mmol/L)	297	1.98 (0.97)	1.99 (1.02)	1.94 (0.89)	2 (0.96)

### Risk factor assessment for sperm quality

**Fig. 1:**
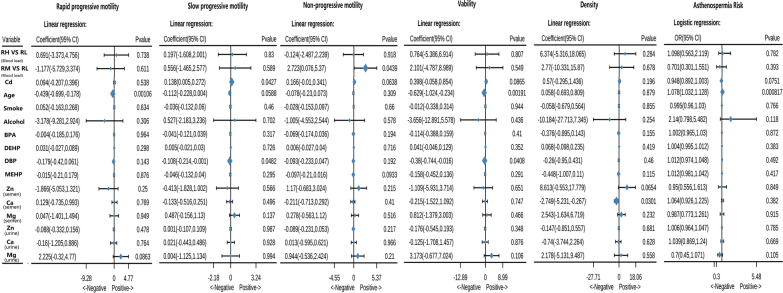
Forest plot showing adjusted coefficient or odds ratio (OR) and 95% confidence interval (CI) for semen parameters in the low Pb exposure group. (RL: 2.5 µL/dL  ≥  Blood lead level  ≥  0 µg/dL; RL: 5 µL/dL  ≥  Blood lead level  >  2.5 µg/dL; RL: 10 µL/dL  ≥  Blood lead level  >  5 µg/dL)

A preliminary multiple regression model was used to analyze the association between BLL and semen parameters. The non-progressive motility was significantly increased in the RH group compared with the RL group [coefficient: 2.72, 95% confidence interval (CI) 0.075–5.37; *p * =  0.044). However, no significant association was observed between Pb exposure and the proportion of rapid progressive motility, slow progressive motility, sperm viability, sperm density, and risk for asthenospermia after adjusting for age, essential metal elements, plastifiers, and smoking or alcohol status (Fig. 1). Non-progressive motility is the inability of the sperm to swim efficiently toward the egg and is considered a risk factor of asthenospermia. Our findings suggest that BLL in the range of 5–10 µg/dL is also an independent risk factor for poor sperm quality.

### MeDIP and hMeDIP sequencing of sperm DNA exposed to low Pb level

**Fig. 2:**
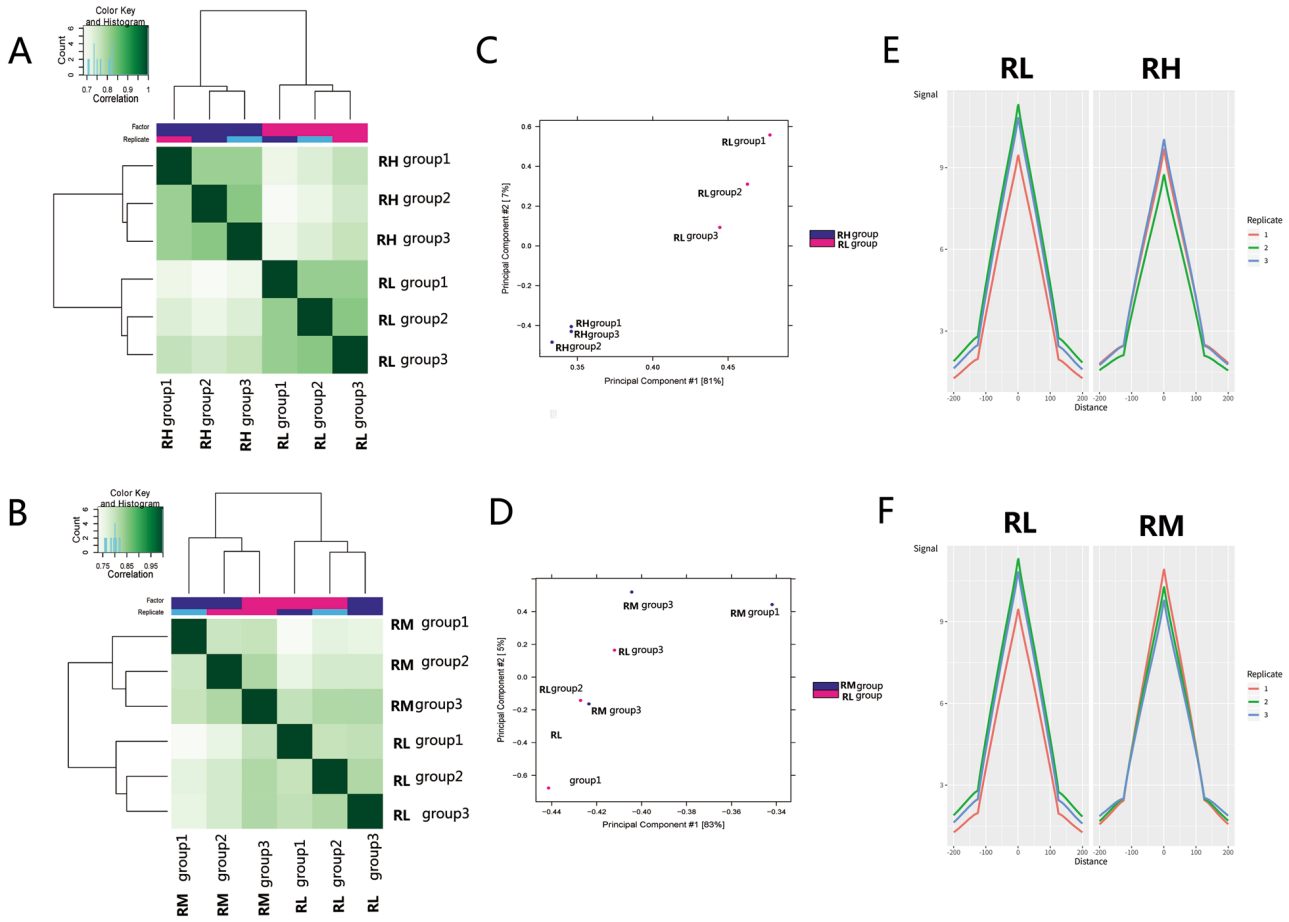
5mC methylation partners in different environments Pb exposure groups. **A** Correlation heatmap showing 5 mC methylation correlation between the RH group (blood Pb, 5–10 µg/dL) and the RL group (blood Pb, 0–2.5 µg/dL). **B** Correlation heatmap showing 5 mC methylation correlation between the RM group (blood Pb, 2.5–5 µg/dL) and RL group (blood Pb, 0–2.5 µg/dL). **C** Principal component analysis (PCA) of methylation measurements in the RH and RL groups. **D** PCA of methylation measurements in the RM and RL groups. **E**Comparison of the average coverage profile at  ±  200 bp near the 5 mC methylation peaks summit between the RH and RL groups. **F** Comparison of the average coverage profile at  ±  200 bp near the 5 mC methylation peaks summit between the RM and RL groups

To determine whether the poor sperm quality is due to low-level Pb exposure-induced impaired DNA methylation, two important methylation forms of sperm DNA, namely 5-methylcytosine (mC) and 5-hydroxymethylcytosine (hmC), were assessed using the MeDIP and hMeDIP sequencing. Approximately 20 million reads were generated for each sample, and the uniquely aligned ratio was about 70% (Additional file 1: Table S1). Hierarchical clustering analysis indicated extremely different sperm DNA methylation patterns in the RH and RL groups. The RM group did not display a considerable difference in the DNA methylation pattern compared with the RL group (Fig. 2A, B). According to the principal component analysis (PCA), PC1 could explain  >  80% methylation variation. Difference between the RH and RL groups in the PC1 direction was observed; however, no obvious difference in the PC1 was observed between RM and RL groups (Fig. 2C, D). We also evaluated the average signal profile across peaks, and the results revealed that the 5mC signal in the RH group decreases globally compared with the RL group and that the sperm methylation profile in the RM group is not obviously different from that in the RL group (Fig. 2E, F). These findings suggest that global sperm DNA hypomethylation induced by Pb exposure may be associated with an increased proportion of sperms with non-progressive motility. We also analyzed the distribution of different hydroxymethylation regions between the RH and RL groups. Additional file 7: Figure S1A, B indicate no obvious difference in the hydroxymethylation signal and profile between the RH and RL groups.

### Distribution of differentially methylated regions and hydroxymethylated regions

**Fig. 3:**
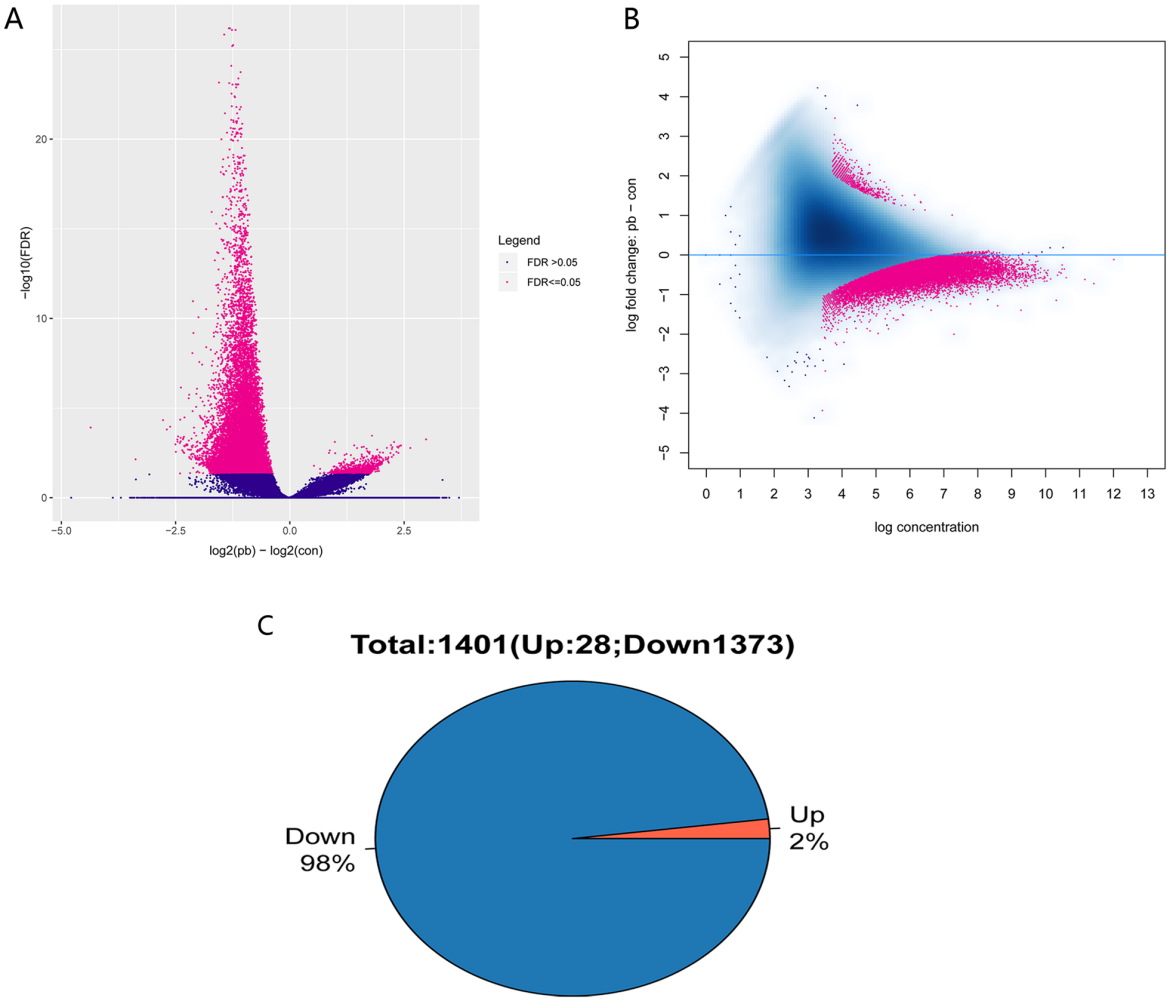
**A** Volcano plot showing differentially methylated regions between the RH and RL groups. **B** MA plots of the log average of the methylation levels on the X-axis and log ratio of the methylation levels on the Y-axis, RH and RL groups. **C** Pie charts depicting genes with differential methylation in the promoter regions

MACS2 was used to identify the distribution of different methylation regions between the RH and RL groups. A total of 18,259 significantly different peaks were identified in the whole genome, of which 17,558 peaks were downregulated and 701 were upregulated (Fig. 3A; Additional file 2: Table S2). These differential peaks had a high reads alignment (Fig. 3B). The 5 mC methylation of the promoter region can cause a low expression of related genes. We screened 1401 genes (1373 hypomethylation genes and 28 hypermethylation genes; Fig. 3C, Additional file 3: Table S3) related to environmental Pb exposure.The promoter regions of these genes contain at least one differentially methylated peaks. Because the methylation variation between the RM and RL groups was small, we found only one significant differential peak (data not shown). Finally, we obtained 12 differentiated peaks, and interestingly, most of them were found to be located on the Y chromosome (Additional file 8: Figure S2).

### Enrichment analysis of DMR-related genes

**Fig. 4:**
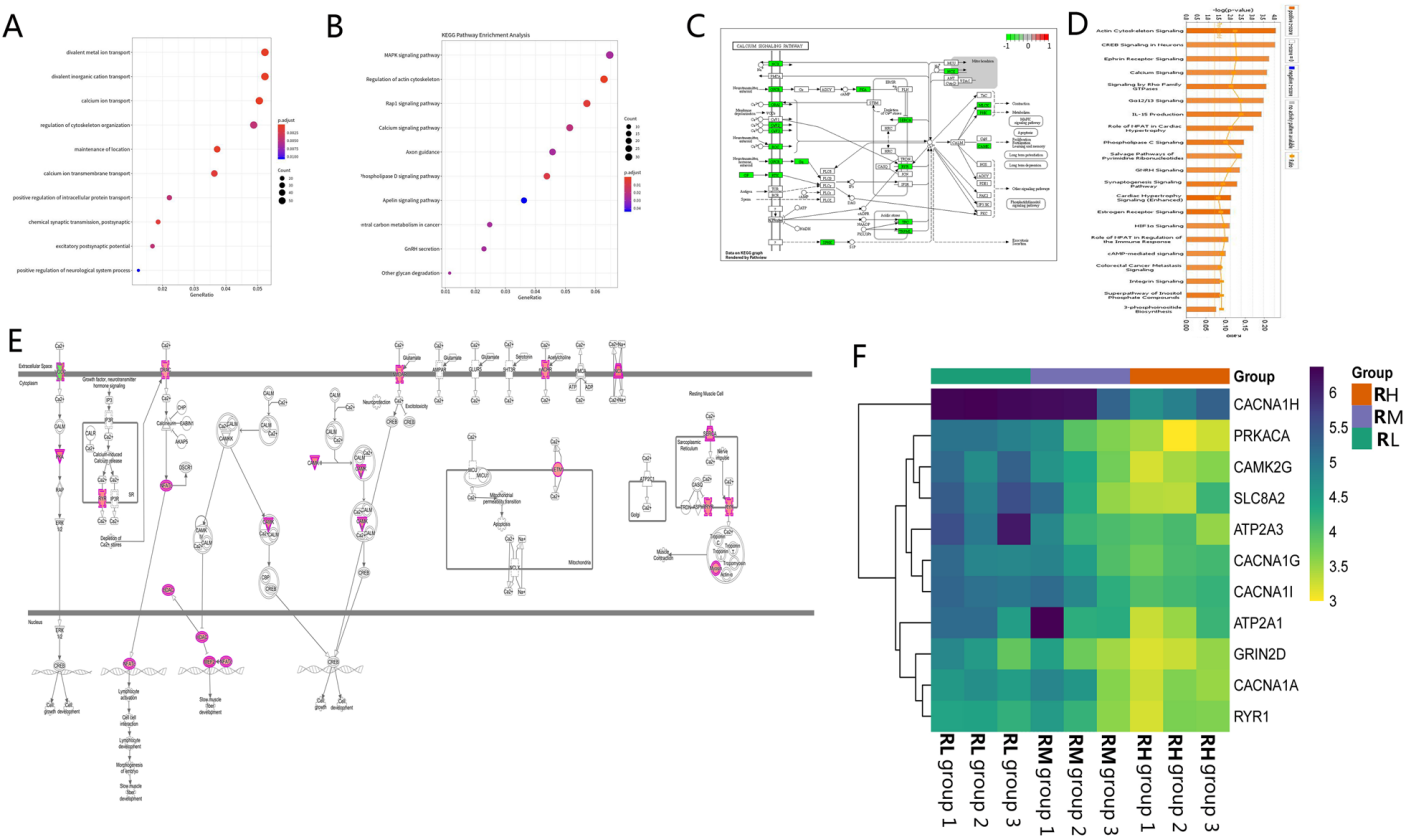
**A** Gene Ontology enrichment analysis for biological processes. **B** KEGG signaling pathways analysis. **C** Visualization of the previously determined calcium signaling pathway using the Pathview package based on KEGG pathways. **D** Ingenuity pathway analysis (IPA) of differentially regulated genes. **E** Visualization of genes enriched in the calcium signaling pathway in IPA analysis. **F** Heatmap summarizing significantly differentially methylated genes enriched in the calcium homeostasis pathway in all three databases (GO, KEGG, and IPA)

We performed the enrichment analysis of differential genes. The enriched GO terms were related to biological processes, as shown in Fig. 4A; Additional file 4: Table S4. Exposure to Pb seemed to alter several pathways in the KEGG enrichment analysis, as is shown in Fig. 4B; Additional file 5: Table S5. Genes with methylation differences in the promoter regions were submitted to the IPA analysis. The analysis showed that differentially methylated genes are mainly related to the neurological system, and cytoskeleton and Ca pathways (Fig. 4D; Additional file 6: Table S6), in agreement with the enriched terms identified using the GO and KEGG. Moreover, the promoter regions of the Ca pathway genes enriched in GO, KEGG, and IPA were all hypomethylated. We further tried to understand the biological relevance of these genes associated with differentially methylated regions (DMRs) in the Ca pathway homeostasis regulation.

Pathway analysis revealed 17 genes with promoter hypomethylation in the KEGG Ca signaling pathway and 15 genes with promoter hypomethylation in the IPA Ca signaling pathway (Fig. 4C, E). Eleven differentially methylated genes simultaneously enriched in the GO (Ca ion transport), KEGG (Ca signaling pathway), and IPA (Ca signaling pathway) were highlighted in the heatmap (Fig. 4F). These findings indicate that the mechanism of Pb toxicity is not only limited to essential metals and metal-dependent biological enzymes but also affects the methylation level of promoters related to the Ca homeostasis pathway genes.

### Effect modification of Ca and Pb on sperm motility

Although no significant association of low Pb exposure or Ca concentration with sperm progressive motility and density was observed in the preliminary model, DNA methylation results showed a close association between Pb and Ca homeostasis pathways, even in the population with low Pb exposure. Therefore, we further evaluated the presence of interactions between Pb and Ca in these individuals. A test for interaction revealed a two-way effect modification between Pb exposure and seminal Ca concentration for predicting rapid progressive motility. However, this effect modification was found only in the RM group (coefficient: 1.2, 95% CI 0.12–2.2; *p * =  0.029) compared with the RL group. Results of the nested model test indicated that the full model that involves the interaction effect is superior to the preliminary model in predicting rapid progressive motility of sperms (Table 2; *p*  =  0.057). A relationship between low Pb exposure and sperm motility was not observed in the preliminary model possibly because the interaction between Ca and Pb masked the influence of Pb exposure on sperm motility. We also did not observe a significant interaction between urinary Ca and low Pb exposure, and therefore, we excluded it from the full model (data not shown). In the full model of other semen parameters, we did not observe a significant interaction between Pb and Cd.

**Table 2:** Effect modification of seminal calcium and Pb of sperm quality and nested model comparisons

	RM vs RL	RH vs RL	splCa	(RM vs RL): splCa	(RH vs RL): splCa
Rapid progressive motility					
Estimate	− 10	− 0.021	− 0.2	1.2	− 0.096
p value	0.056	0.99	0.68	0.029	0.87
95% CI	− 20 to 0.28	-11 ~ 11	− 1.2 to 0.76	0.12 to − 2.2	− 1.2 to 1.1
p value of nested model test	0.057				
Slow progressive motility					
Estimate	− 2.4	− 1.9	− 0.27	0.28	0.26
p value	0.32	0.49	0.23	0.24	0.32
95% CI	− 7.1 to 2.4	− 7.16 to 3.42	− 0.70 to 0.16	− 0.18 to − 0.74	− 0.25 to 0.77
p value of nested model test	0.41				
Non-progressive motility					
Estimate	− 0.35	4	− 0.19	0.02	− 0.14
p value	0.91	0.25	0.51	0.95	0.69
95% CI	− 6.6 to 5.9	− 2.9 to 11	− 0.76 to 0.37	− 0.59 to − 0.63	− 0.81 to 0.54
p value of nested model test	0.9				
Sperm concentration					
Estimate	8.6	21	− 2.3	− 0.28	− 2
p value	0.58	0.22	0.11	0.86	0.25
95% CI	− 22 to 39	− 13 to 56	− 5.1 to 0.49	− 3.3 to − 2.7	− 5.3 to 1.4
p value of nested model test	0.5				
Sperm viability					
Estimate	− 9.3	2.5	− 0.37	1.1	− 0.021
p value	0.29	0.83	0.63	0.18	0.99
95% CI	− 27 to 8.2	− 20 to 25	− 1.9 to 1.1	− 0.55 to − 2.8	− 2.4 to 2.3
p value of nested model test	0.38				
Asthenozoospermia					
OR	6.2	0.27	1.1	0.83	1.1
p value	0.05	0.22	0.2	0.055	0.34
95% CI	− 22 to 39	− 13 to 56	− 5.1 to 0.49	− 3.3 to − 2.7	− 5.3 to 1.4
p value of nested model test	0.03				

Furthermore, we analyzed whether low Pb exposure is related to the risk of asthenospermia, as stated in the fifth edition of the WHO guidelines. The results demonstrated that there is a two-way effect modification between Pb exposure and seminal Ca concentration for predicting asthenospermia, and the effect was found to exist only in the RM group when compared with the RL group (OR: 0.83, 95% CI 0.68–0.99; *p * =  0.05). Results of the nested model test supported the superiority of the full model that involves the interaction effect to the preliminary model (*p*  =  0.030; Table 2).

## Discussion

Our findings showed that a BLL of 5–10 µg/dL can still reduce the sperm motility in men of childbearing age, which is inconsistent with the results of a previous Mexican cohort study [23]. The primary reason for this difference may be the lack in previous studies of assessment of other risk factors, such as Cd, trace elements, or plastifiers, that are associated with male reproductive toxicity. Unlike a recent study in Southwest China, which reported that low Pb exposure adversely effects the sperm movement and density, our research shows that low Pb exposure is related only to the sperm motility. The difference may be that the individuals with BLL  >  10 µg/dL were not excluded in the study in Southwest China [24].

Majority of studies have focused on determining the effects of environmental Pb exposure on DNA methylation in human peripheral blood cells [9, 25, 26]. Moreover, analysis in some studies that focused on determining the epigenetic effects of Pb exposure on the male reproductive system had been limited to sperm DNA damage and changes in the sperm chromatin [23, 27]. Our research confirms that the effect of BLL as low as 5 µg/dL on decreased sperm motility can be similar to that of the occupational Pb exposure. We found that low Pb exposure (BLL of 5–10 vs 0–2.5 µg/dL) changes the DNA methylation pattern of human sperm and causes global hypomethylation, which established the mechanism linking the decreased sperm motility with low Pb exposure and DNA methylation. However, we did not observe a global change in sperm DNA methylation in the 2.5–5 µg/dL group. The finding indicates that the effect of Pb exposure on sperm DNA methylation is dose-dependent and that a BLL of 5 µg/dL could be used as a threshold value to indicate the safety of DNA methylation in male gametes. In addition, we detected the DNA hydroxymethylation level in the RH and RL groups and observed no global difference in the sperm DNA hydroxymethylation, which imply that hydroxymethylation may be insensitive to low Pb exposure. Compared with other studies, this study expands the perception of the effect of environmental Pb exposure on DNA methylation of male gametes [28, 29].

We also identified 1401 differentially methylated genes (DMGs) in promoter regions. Among them, the promoter regions of 1373 genes were hypomethylated, suggesting that environmental Pb exposure may be associated with abnormally high expression of genes in sperm cells. This finding is consistent with a study that reported a correlation between Pb exposure and decreased DNA methylation [30]. We analyzed the enrichment of these DMGs through IPA, GO, and KEGG analysis. Results were consistent and indicated that the functions of DMGs are mainly enriched in the cytoskeleton and neurological system pathways. Abnormal cytoskeleton has been reported to be an important factor for sperm motility [31]. Our result confirms that the reduced sperm motility reduction is associated with the DNA methylation pattern caused by low Pb exposure. Moreover, significant enrichment of DMGs in the neurological system necessitates further study to understand the association between paternal Pb exposure and offspring nervous system disorders [32].

Our findings revealed that low Pb exposure causes hypomethylation and hence impairs the Ca homeostasis pathway genes in sperm cells. However, in the preliminary model, we did not observe a significant correlation between seminal Ca or urinary Ca and sperm progressive motility. Previous studies have reported an antagonistic interaction between Pb and Ca; therefore, Ca supplementation is considered an effective antidote for chronic Pb poisoning [33–35]. We assume that Ca influx may antagonize the effect of low Pb exposure on sperm motility. In the full model with the interaction factors of blood Pb and seminal Ca, we observed statistically significant modification effects of low Pb exposure and seminal Ca on the rapid progressive motility of sperms and risk of asthenospermia as well.

Genomic regulation through epigenetic modifications in Ca homeostasis-related pathways is an important mechanism to maintain human health [11]. Our research showed that Ca may be a protective factor for men of childbearing age with a BLL of  <  5 µg/dL and may help to avoid the deterioration of sperm motility induced by Pb exposure. However, in those with a BLL of  >  5 µg/dL, we observed no significant interaction between Pb and Ca accompanied by abnormal methylation of Ca homeostasis regulatory genes in rapid progressive motility sperm cells. This finding suggests that the interaction between Ca and Pb is epigenetically controlled under the condition of low Pb exposure. Moreover, Ca does not protect against abnormal DNA methylation caused by high Pb exposure beyond 5 µg/dL. The potential reason may be Pb exposure beyond 5 µg/dL interfered methylation-dependent Ca homeostasis regulation. Similarly, we did not observe the interaction between Pb and Ca in non-progressive motility sperm cells which have been reported carrying inherent methylation abnormalities [36]. This potential mechanism may account for the ineffectiveness of Ca supplementation in alleviating Pb poisoning.

Our study has certain limitations. The results must be interpreted in the context of the Chinese population and limitations inherent to human data. Moreover, although data were adjusted for multiple known confounding factors such as age, smoking and alcohol status, endocrine disruptors, and Cd, individuals are typically exposed to factors, such as chemical stressors (exposure to nearly 200 chemicals) and nonchemical stressors (anxiety and stress), that can affect the sperm quality. We, therefore, cannot exclude the possibility that the reported results may be related to other exposure factors. These limitations notwithstanding our data suggest that Pb exposure is associated with epigenetic perturbations, including Ca pathway gene hypomethylation, which has been linked to various diseases. Currently, another cohort study in the eastern China is investigating the impact of environmental Pb exposure on the Ca pathway.

## Supplementary Information

**Additional file 1: Table S1.** Reads alignment ratio.

**Additional file 2: Table S2.** Significant differential 5 mC peaks between the RH and RL groups.

**Additional file 3: Table S3.** Genes differentially 5 mC methylated in the promoter regions between the RH and RL groups.

**Additional file 4: Table S4.** Gene Ontology analysis result.

**Additional file 5: Table S5.** Kyoto Encyclopedia of Genes and Genomes (KEGG) analysis results.

**Additional file 6: Table S6.** Ingenuity pathway analysis results.

**Additional file 7: Figure S1.** (A) Correlation heatmap showing the 5hmC correlation between the RH and RL groups. (B) Principal component analysis (PCA) of 5hmC measurements in the RH and RL groups. (C)Comparison of the average coverage profile at ±200 bp near 5hmC peaks between the RH and RL groups.

**Additional file 8: Figure S2.** Heatmap summarizing the significant differential hydroxymethylcytosine regions between the RH and RL groups.

## Data Availability

All underlying data are provided in the manuscript or available upon request.

## Abbreviations

BLL: Blood lead level
NP: Non-progressive motility
MeDIP: Methylated DNA immunoprecipitation
hMeDIP: Hydroxymethylated DNA immunoprecipitation
FSH: Follicle stimulating hormone
LH: Luteinizing hormone
DEHP: Dioctyl phthalate
BPA: Bisphenol A
DBP: Dibutyl phthalate
MEHP: Mono-2-ethylhexyl phthalate
DMGs: Differentially methylated genes
