# Supplementary figures and images for: Effects of low lead exposure on sperm quality and sperm DNA methylation in adult men
Source: Cell Biosci. 2021 Jul 30;11:150. doi: 10.1186/s13578-021-00665-7 (PMC8335892; doi:10.1186/s13578-021-00665-7)

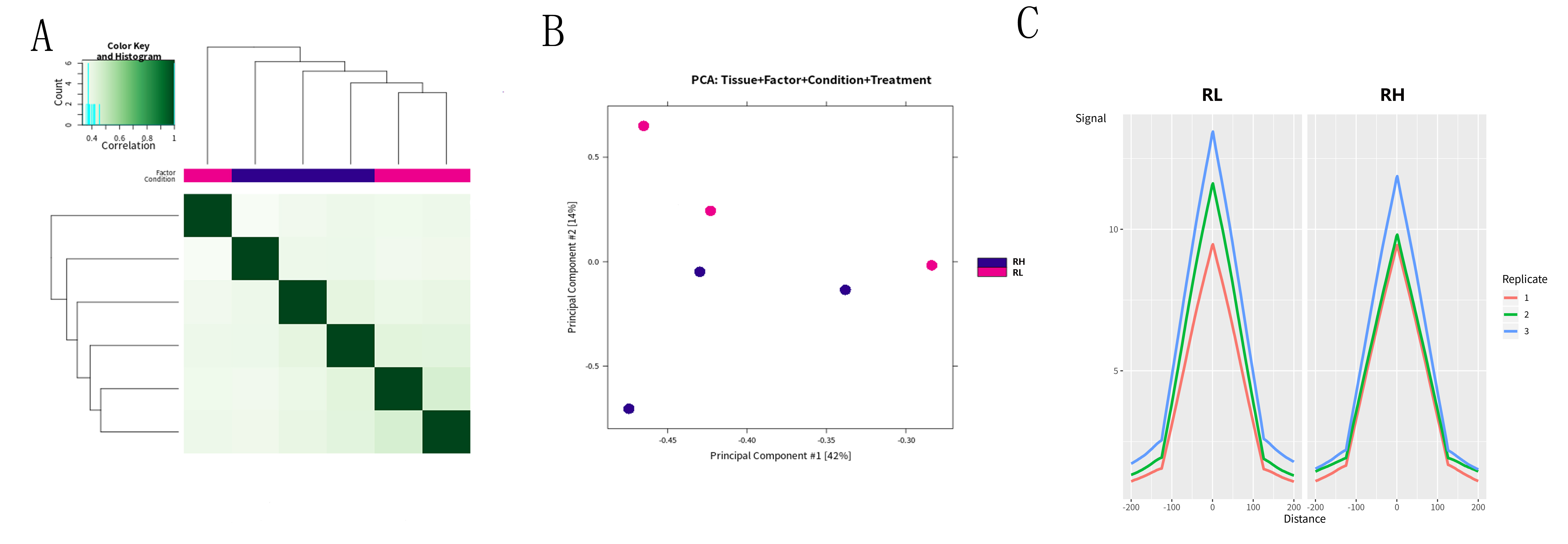

Supplement: Supplementary file 7 — Additional file 7: Figure S1. (A) Correlation heatmap showing the 5hmC correlation between the RH and RL groups. (B) Principal component analysis (PCA) of 5hmC measurements in the RH and RL groups. (C)Comparison of the average coverage profile at ±200 bp near 5hmC peaks between the RH and RL groups. [file 13578_2021_665_MOESM7_ESM.jpg]

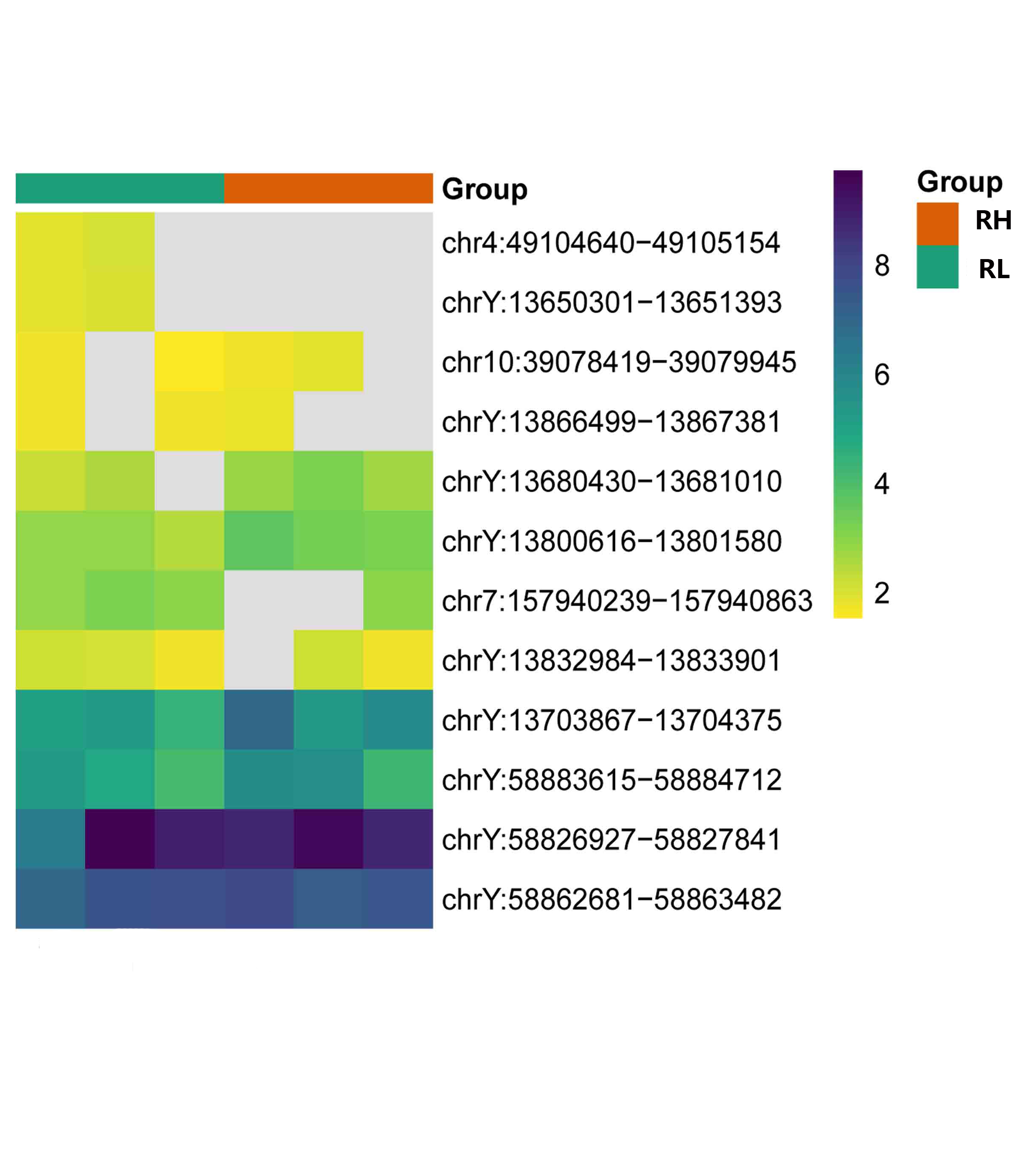

Supplement: Supplementary file 8 — Additional file 8: Figure S2. Heatmap summarizing the significant differential hydroxymethylcytosine regions between the RH and RL groups. [file 13578_2021_665_MOESM8_ESM.jpg]
